# Massively parallel characterization of adolescent idiopathic scoliosis risk variants

**DOI:** 10.1101/gr.281888.126

**Published:** 2026-10

**Authors:** Darius Ramkhalawan, Justin Koesterich, Fahim Rejanur Tasin, Carlos Cuna, Anat Kreimer, Nadja Makki

**Affiliations:** 1 Department of Physiology and Aging, University of Florida, College of Medicine, Gainesville, Florida 32610, USA;; 2 Graduate Programs in Molecular Biosciences, Rutgers, The State University of New Jersey, Piscataway, New Jersey 08854, USA;; 3 Department of Biochemistry and Molecular Biology, Rutgers, The State University of New Jersey, Piscataway, New Jersey 08854, USA;; 4 Center for Advanced Biotechnology and Medicine, Rutgers, The State University of New Jersey, Piscataway, New Jersey 08854, USA;; 5 Genetics Institute, University of Florida, Gainesville, Florida 32610, USA

## Abstract

Adolescent idiopathic scoliosis (AIS) is a common pediatric musculoskeletal disorder characterized by lateral spinal curvature, often leading to chronic pain and deformity. Although a significant genetic component to AIS is recognized, the functional impact of most associated genetic variants, particularly those in noncoding regions, remains largely unknown. Using massively parallel reporter assays, we characterize 1664 variant positions in linkage disequilibrium with 26 AIS lead variants identified by genome-wide association studies (GWASs) in chondrocytes, a major cell type implicated in AIS pathogenesis. Using a library of 7173 candidate regulatory sequences, we compare the 1664 reference alleles against 4708 alternate alleles in two human chondrocyte cell lines (TC28a2 and SW1353). Our analysis identifies 92 variants that exhibit significant differential regulatory activity between their reference and alternate alleles, 79 of which are predicted to disrupt transcription factor binding sites, often correlating with their observed regulatory effect. Notably, we validate rs9496392, a single-nucleotide variant near the *ADGRG6* locus, which shows consistent differential regulatory activity in both cell lines. *ADGRG6* is a key regulator of cartilage homeostasis, and its cartilage-specific knockout in mice results in a scoliosis-like phenotype. The AIS risk allele of rs9496392 (T) is predicted to strongly disrupt several TFBSs, including SP1. This study provides a foundational catalog of functional AIS-associated regulatory variants active in chondrocytes, offering crucial insights into the perturbed gene regulatory networks in AIS. These findings lay the groundwork for identifying biomarkers and potential therapeutic targets for this complex childhood disease.

Adolescent idiopathic scoliosis (AIS), a lateral spinal curvature spontaneously developing during puberty in otherwise healthy children, is the most common pediatric musculoskeletal disorder globally (Rogala et al. 1978; Wise et al. 2008; Weinstein 2019). The progression of AIS can severely impact a patient's life, leading to chronic pain, physical deformity, spinal osteoarthritis, and impaired lung and heart function (Hoelen et al. 2023). Current treatment options are limited to bracing or corrective surgery, and the absence of presymptomatic diagnostic capabilities underscores the need for a deeper mechanistic understanding of the disease.

Genetic studies have established a significant genetic component to AIS (Andersen et al. 2007; Tang et al. 2012; Yang et al. 2012; Grauers et al. 2013; Buchan et al. 2014a; Karner et al. 2015; Ogura et al. 2015; Patten et al. 2015; Haller et al. 2016; Blecher et al. 2017; Yonezawa et al. 2020; Gray et al. 2021; Liu et al. 2021; Ushiki et al. 2024; Tuncay et al. 2025), with numerous susceptibility loci identified through genome-wide association studies (GWASs) (Sharma et al. 2011; Takahashi et al. 2011; Fan et al. 2012; Kou et al. 2013, 2019; Miyake et al. 2013; Londono et al. 2014; Ogura et al. 2015, 2017; Sharma et al. 2015; Sudmant et al. 2015; Zhu et al. 2015; Khanshour et al. 2018; Kou et al. 2018; Wu et al. 2019; Wise et al. 2020; Yu et al. 2024). However, the vast number of potential causal variants, especially those in linkage disequilibrium (LD) with GWAS hits (exceeding 1000), presents a major hurdle. Most of these variants are located in noncoding regions, suggesting their involvement in regulating gene activity. A few studies have begun to identify AIS-associated variants with altered regulatory function through individual enhancer assays (Sharma et al. 2015; Yonezawa et al. 2020). It was also recently demonstrated that knockout of two enhancer elements harboring AIS-associated variants at the *PAX1* locus results in a kinked tail phenotype in mice (Ushiki et al. 2024). However, the comprehensive identification of functional variants at AIS loci remains an outstanding challenge.

Compelling evidence supports the involvement of abnormal cartilage biogenesis and development in AIS onset. Recent studies suggest that estrogen signaling may disrupt extracellular matrix homeostasis in growth plate cartilage (Yu et al. 2024), and cartilage-specific deletion of the AIS susceptibility gene *ADGRG6* in mice has been shown to induce a scoliotic curve during adolescence (Karner et al. 2015; Liu et al. 2021). Despite these insights from human genetic studies and animal models, a critical gap exists in understanding how the non-coding variants identified in patients contribute mechanistically to AIS pathogenesis.

In this study, we carried out the first massively parallel reporter assay (MPRA) to comprehensively assess the regulatory activity of AIS-associated variants. This innovative, high-throughput approach allowed for an unbiased examination of all AIS GWAS variants (Wise et al. 2020) and those in LD in a single quantitative experiment. Given the central role of chondrocytes in AIS pathogenesis, MPRAs were performed in two human chondrocyte cell lines. This work provides a highly confident list of functional variants and their regulatory effects. The functional variants identified in this study will serve as a foundational resource for elucidating altered gene regulatory networks in AIS patients and will significantly advance the future investigations of this common childhood disease. Importantly, this work lays the groundwork for the identification of AIS biomarkers and potential drug targets.

## Results

### MPRA variant library design and selection of cell lines

We designed our MPRA library to include all 26 lead AIS GWAS SNPs (Wise et al. 2020), as well as variants in LD (*r*^2^ ≥ 0.7) in European, Han Chinese, and ethnic Japanese populations, resulting in 1664 unique variants (1522 SNPs, 142 insertions and deletions [indels]) (Supplemental Table S1). Each candidate regulatory sequence (CRS) was generated as a 200 bp sequence with the variant of interest at base pair 101. For each variant, we included all possible alleles (four per SNP, two per indel), resulting in a total of 6372 test CRSs. Given the central role of cartilage in AIS pathogenesis (Karner et al. 2015; Liu et al. 2021), we sought to conduct our MPRA in two commonly used human chondrocyte cell lines: TC28a2 and SW1353. For positive controls, we identified regions of significant H3K27ac enrichment and filtered peaks according to RNA-seq expression in both cell lines, in which only sequences within 1 Mb of a gene with expression of transcripts per million (TPM) > 1 were retained, resulting in 601 positive control sequences. As negative controls, we included 200 scrambled sequences from randomly selected reference CRSs. This resulted in a total library size of 7173 CRSs, including those with AIS variants to be tested for regulatory activity, as well as positive and negative controls (Fig. 1).

**Figure 1.**
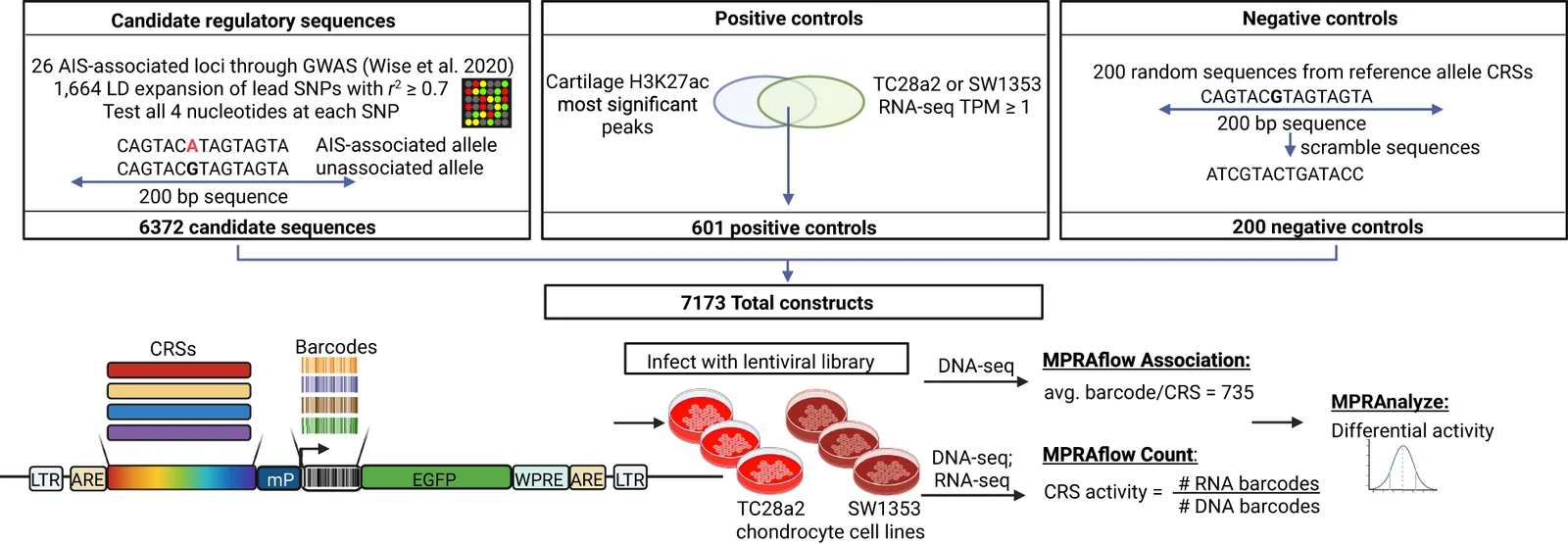
Overview of the MPRA design. This schematic shows the selection process of choosing the candidate regulatory sequences to be investigated in the assay (*top left*), selection of positive controls (*top middle*), and negative controls (*top right*). *Bottom* panel shows an overview of the MPRA steps, including the segment of DNA that is integrated into the cell genome (*left*), transduction into chondrocyte cell lines (*middle*), and the downstream sequencing and processing of the MPRA (*right*).

### MPRA association, count, and significant variant pairs

A minimal promoter, random barcode sequences, and vector overhangs were added to CRS oligonucleotides by sequential rounds of PCR and cloned into a lentiMPRA reporter vector. To associate CRSs with their respective barcodes, we sequenced the CRS-barcode fragment and utilized the MPRAflow pipeline to associate the randomly combined transcribable barcodes with the upstream CRS (Gordon et al. 2020). We identify more than 7.3 million barcodes confidently assigned to our CRSs at an average of 735 barcodes per CRS.

Next, we infected each cell line with the lentivirus library in triplicate and then simultaneously isolated DNA and RNA for sequencing of DNA and RNA barcodes. Active CRSs drive transcription of the downstream barcode sequences; the transcribed RNA barcodes are then normalized to the integrated DNA barcodes to quantify the gene regulatory activity of the CRS.

We analyzed the DNA and RNA obtained from the infected chondrocytes and matched the counts for the barcodes with those that were associated with the CRSs. We were able to match counts for 66.27% of the confidently associated barcodes in the TC28a2 cell line. We observed an average *r* correlation value of 85% for the DNA barcode counts between replicates and 80.33% for RNA barcode counts between replicates (Supplemental Fig. S1). For the SW1353 cell line, we confidently associated 61.98% of barcodes and observed an average *r* correlation value of 87.33% for the DNA barcode counts between replicates and 77% for RNA barcode counts between replicates (Supplemental Fig. S2). When aggregating replicate counts to the CRS level, we observe ∼98% DNA, 96% RNA, and 92% RNA/DNA ratio *r* correlation values between replicates in both cell lines (Supplemental Figs. S3 and S4).

After quality-control steps were performed, we retained 6266 CRSs for TC28a2 and 6265 CRSs for SW1353, plus 791 positive and negative controls, for further statistical analysis, with a total of 4,878,602 and 4,563,151 confidently counted barcodes, for TC28a2 and SW1353 respectively. Next, we utilized the MPRAnalyze program (Ashuach et al. 2019) to generate estimated transcription rates from the sequenced RNA and DNA. These transcription rates, referred to as alpha values, are first compared to the negative controls to identify sequences that are significantly active in the cell line. We identified a total of 1566 CRSs in TC28a2, 911 in SW1353, and 2008 combined across both cell lines (659, 499, and 886 unique loci, respectively) that had a significantly active transcription rate compared with the negative controls (*P*-value ≤ 0.05) (Fig. 2A,B; Supplemental Tables S2, S3). This represents the first functional high-throughput identification of active enhancer elements in chondrocytes and will serve as a new catalog of active enhancers in TC28a2 and SW1353 cells.

**Figure 2.**
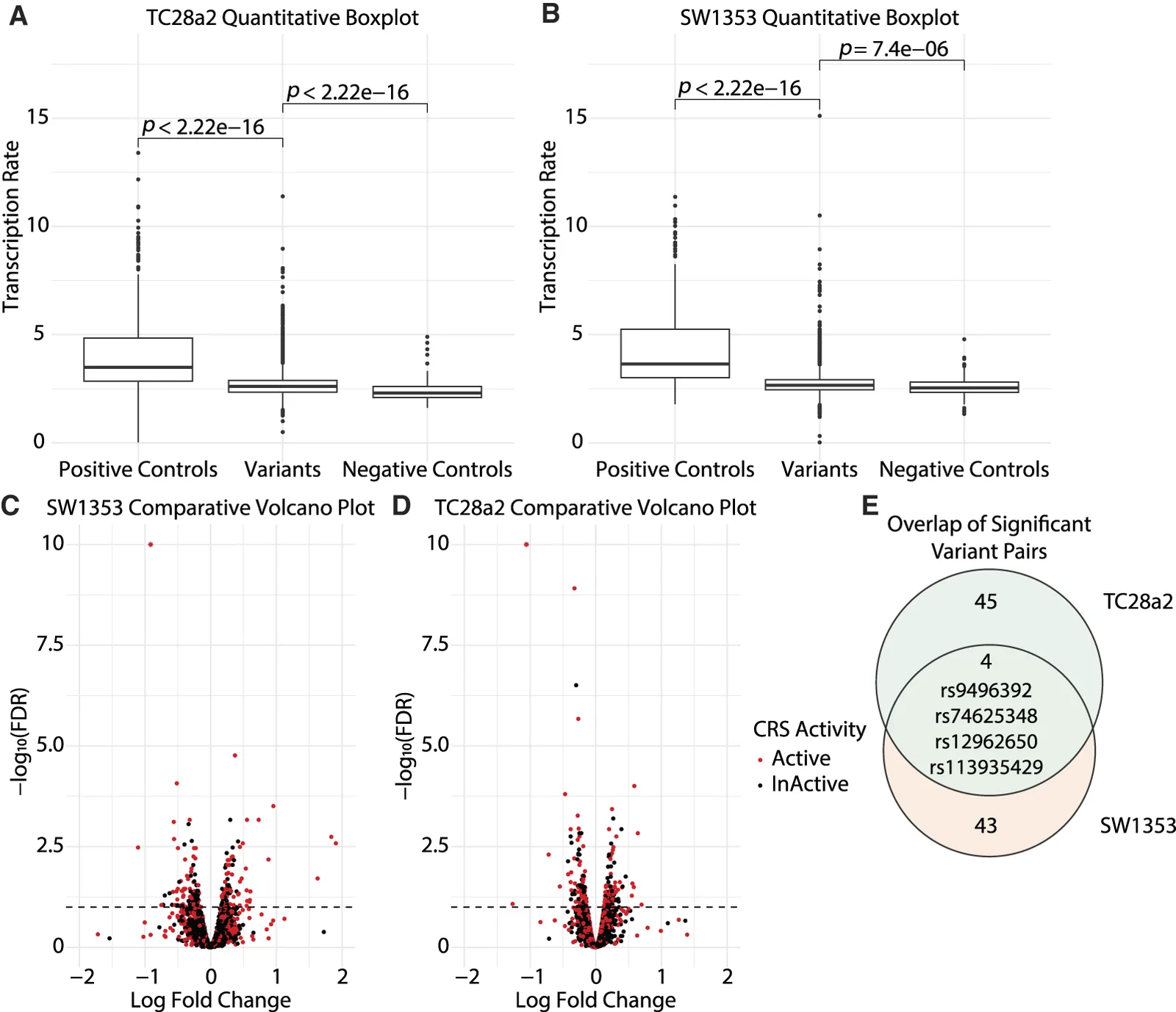
Quantitative and comparative MPRA results. (*A*,*B*) Boxplots showcasing the quantitative analysis performed on the RNA/DNA transcription rate for all CRSs, separated into positive controls, negative controls, and combined reference and alternate allele (variants). *P*-values at the *top* of the boxplots were calculated using the Wilcoxon rank-sum test comparing the median transcription rate between the two conditions listed under the bracket. A separate *P*-value calculation is done by MPRAnalyze to determine individual CRSs that are active compared to the negative controls (Methods). (*C*,*D*) Volcano plots showing the comparative analysis performed by MPRAnalyze on the reference alternate variant pairs. Each point is a single reference/alternate variant pair, positioned based on the comparative analysis and colored based on the quantitative analysis. The *x*-axis denotes the natural log fold change values of the CRS; a positive LFC indicates a higher transcription rate in the reference allele compared with the alternate allele. The *y*-axis denotes the –log_10_ of the FDR value that is associated with the variant pair's log fold change. A variant pair is called as significantly disruptive if the FDR value is less than 0.10, denoted by the dashed line. Additionally, we call a final significantly disruptive variant pair list as those that are both significantly disruptive, *above* the dashed line, and with either the reference or alternate allele significantly active compared with the negative controls (denoted as red). (*E*) Overlap of the final significant observed alternate allele variant pairs identified in the two cell lines.

MPRAnalyze then groups the CRSs by reference–alternate variant pairs, which are analyzed to identify variant pairs that contain a significant difference in transcription between the two alleles. As our library contains 1664 unique variants, comparing each SNP reference allele to its three alternate alleles (1522 SNPs × 3 = 4566) plus 142 indel pairs results in 4708 total testable variant pairs. After removing incomplete variant pairs and variant pairs with only zero or missing counts, we investigated the allelic effect of 4468 variant pairs in both cell lines, representing 1579 of the starting 1664 unique variants.

Of the 4468 retained total variants pairs, we identify 208 total variant pairs in the TC28a2 cell line, along with 192 total variant pairs in the SW1353 cell line, that have a significant change in transcription rates between alleles (false-discovery rate [FDR] ≤ 0.1) (Fig. 2C,D; Supplemental Tables S4, S5). These variant pairs included either the GWAS/HapMap alternate variant or a synthetic alternate variant. To identify functional regulatory variants underlying the observed genetic association in the AIS GWAS, we focused our downstream analysis on those variant pairs that included the GWAS/HapMap (referred to as observed) alternate allele. This resulted in 1579 retained observed alternate allele variant pairs, of which 82 were observed alternate variant pairs in the TC28a2 cell line, along with 73 observed alternate variant pairs in the SW1353 cell line, that have significant change in transcription rates between alleles (FDR ≤ 0.1) (Supplemental Tables S4, S5).

We then overlap the two analyses to generate a set of 92 highly confident variant pairs (49 in TC28a2, 47 in SW1353) that have both a significant change in transcription rates between alleles and at least one allele significantly active compared with the negative controls (Fig. 2; Supplemental Tables S6, S7). Interestingly, four variant pairs (rs113935429 (T > –), rs9496392 (T > G), rs74625348 (G > C), rs12962650 (T > G)) were significant in both cell lines (Fig. 2E). Forty-nine additional variant pairs (55.7%) are found to have the same direction of effect on transcription. Twenty-one of these 49 variant pairs are found with at least one allele active in both cell lines, and perhaps being called as differentially nonsignificant in the second cell line is because of insufficient statistical power rather than biological effects (Supplemental Tables S6, S7). These 92 regulatory variants are spread across 18 of the 26 AIS GWAS loci and contain three of the lead SNPs themselves (Supplemental Fig. S5).

### Annotation of significant variant pairs

As enhancers are typically bound by transcription factors (TFs), we next investigated if these variants are predicted to disrupt transcription factor binding sites (TFBS), which would improve our understanding of how specific variants disrupt transcription. Here we leverage the motifbreakR program (Coetzee et al. 2015) to predict differential binding affinity of TFBSs overlapping the variant position. We found that 79 of the 92 variants (85.9%) are predicted to disrupt the binding site of at least one TF that is active in either cell line (Supplemental Tables S6, S7). Of these 79 variant pairs, 59 have a predicted TF disruption in the same direction of effect as the variant pairs’ MPRA log fold change value. This suggests that the variants’ disruption to transcriptional regulation is likely because of their effect on TF binding. Interestingly, one of the variant pairs (rs200323221) (Fig. 3A) is predicted to disrupt the binding of the TF EGR1 (Fig. 3B,C). This variant is shown to have a similar ratio of disruption to both transcription in the MPRA and the TFBS predicted binding (Fig. 3B). EGR1 has previously been associated with AIS, and the disruption of its binding to this potential regulatory region may shed new light on affected transcriptional networks downstream from this TF (Yonezawa et al. 2020).

**Figure 3.**
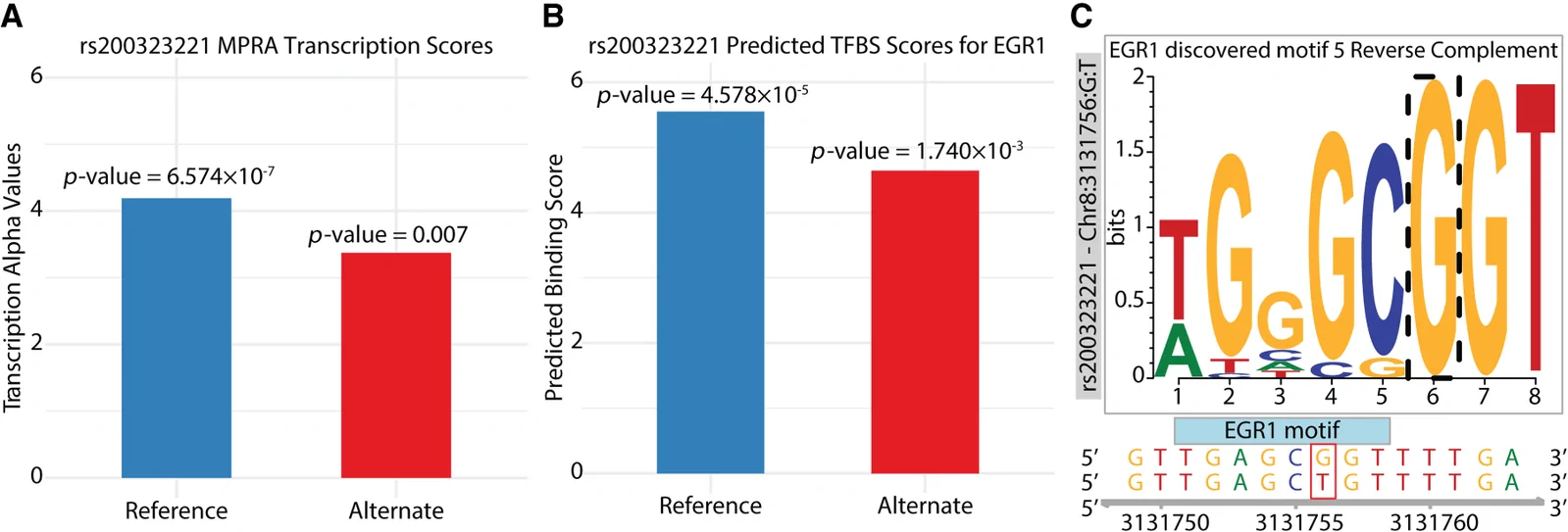
Predicted transcription factor binding site (TFBS) disruptions owing to the variant rs200323221. (*A*) MPRA transcription rate (alpha) value for rs200323221 (G > T) in the SW1353 cell line. The listed *P*-value is an empirical *P*-value reported by MPRAnalyze for significant activity compared with the negative controls in the SW1353 cell line. (*B*) Predicted binding score for the EGR1 motif with the given allele. The listed *P*-values are calculated based on the likelihood of the sequence similarity score of the allele specific sequence to the TFBS compared with the sequence similarity score using the null background allelic distribution. (*C*) LOGO representation of the predicted motif region with the mutated position (dashed box), reference sequence (*below* the graph, *top* row), and the alternate sequence (*bottom* row), with the reference and alternate alleles highlighted in the red-shaded rectangle. The *y*-axis represents the weight of presence each allele has at that position observed in the ENCODE motif database.

Next, we identified genes closest to the 92 significant variants pairs and performed GO (Yu et al. 2012) enrichment analysis. We identify 22 unique proximal genes (Supplemental Tables S6, S7) that were enriched for skeletal system and connective tissue development, cartilage condensation, regulation of cell differentiation, and cell fate specification (Supplemental Fig. S6). When examining expression levels of the 22 genes, we found that nine are expressed in AIS patient cartilage (TPM ≥ 1) (Supplemental Fig. S7; Makki et al. 2021). Next, we examined if any of the genes closest to the 92 variants (Supplemental Tables S6, S7) had previously been linked to AIS through functional studies (Supplemental Table S8; Smits and Lefebvre 2003; Hoornaert et al. 2010; Bachmann-Gagescu et al. 2011; Becker-Heck et al. 2011; Henry et al. 2012; Buchan et al. 2014a,b; Gray et al. 2014; Hayes et al. 2014; Karner et al. 2015; Ogura et al. 2015, 2017; Sharma et al. 2015; Barat-Houari et al. 2016; Grimes et al. 2016; Jaffe et al. 2016; Haller et al. 2018; Van Gennip et al. 2018; Yonezawa et al. 2020; Liu et al. 2021; Su et al. 2021; de Azevedo et al. 2022; Decourtye et al. 2022; Wang et al. 2022, 2023; Bieder et al. 2023; Rebello et al. 2023; Yuet al. 2024; Xu et al. 2025). We identified 40 significant variants in proximity to seven genes previously linked to AIS through functional studies, namely, *ADGRG6*, *BNC2*, *FTO*, *MIR4300HG*, *PAX1*, *SOX6*, and *UNCX*. These findings give us potential insights into the mechanisms of both the variant disruption and the downstream involvement in disease pathology.

### Validation and characterization of rs9496392

Among the 92 significant variant pairs identified in our MPRA (Supplemental Tables S6, S7) was a previously reported regulatory variant, rs169311, located within the PEC7 enhancer proximal to *PAX1*. The risk allele (A) was previously shown to abolish enhancer activity in zebrafish (Sharma et al. 2015), consistent with our MPRA results. We then further validated the regulatory effects of five additional MPRA-identified SNPs using luciferase reporter assays in the TC28a2 cell line (Fig. 4A,B; Supplemental Figs. S8, S9). SNPs were selected based on their proximity to genes with roles in chondrogenesis and cartilage homeostasis (*ADGRG6*, *FTO*, *WNT9A*) (Später et al. 2006; Karner et al. 2015; Shu et al. 2025). Differential luciferase activity of each allele generally coincided with the observed MPRA activity (Fig. 4A,B; Supplemental Figs. S8, S9). Some constructs (rs1040525, rs113935429) showed differential luciferase activity in the opposite direction from the MPRA signal, which could potentially be because of the differing context dependencies that are inherent to the two assays (Inoue et al. 2017; Klein et al. 2019, 2020). One of the validated single-nucleotide variants, rs9496392 (T > G), is located at the *ADGRG6* locus (Fig. 4A,B). ADGRG6 is a key regulator of cartilage homeostasis and helps maintain proper alignment of the vertebral column during postnatal development (Karner et al. 2015). *ADGRG6* is expressed at high levels in human spinal cartilage, mouse chondrocytes, and mouse intervertebral discs (Fig. 4C; Makki et al. 2021). Additionally, *ADGRG6* is significantly downregulated in AIS patient cartilage tissue compared with control samples (Ramkhalawan et al. 2026).

**Figure 4.**
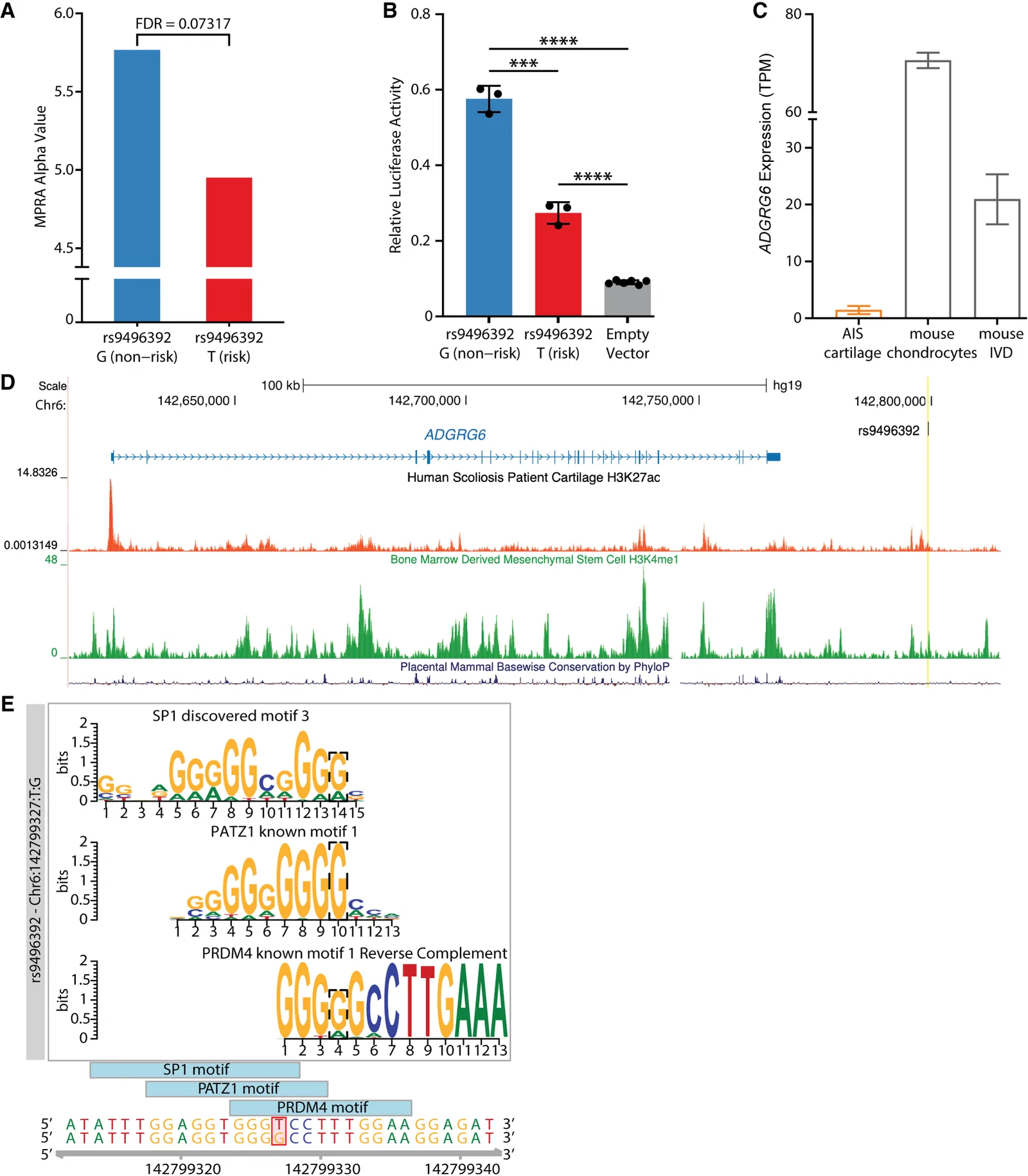
Predicted transcriptional disruption owing to variant rs9496392. (*A*) MPRA signal of rs9496392 CRS in TC28a2 (*n* = 3). The reported false-discovery rate (FDR) value is a FDR adjusted likelihood ratio test *P*-value of the difference in activity between alleles reported by MPRAnalyze. (*B*) Relative luciferase activity of the rs9496392 CRS in TC28a2 (*n* = 3). (***) *P* < 0.001; (****) *P* < 0.0001. The statistical significance between CRS activity and empty-vector negative controls was determined by one-way ANOVA; the statistical significance between SNP alleles was determined by unpaired Student's *t*-test. (*C*) Expression of *ADGRG6* in AIS spinal cartilage, mouse chondrocytes, and mouse intervertebral disc. (*D*) Scoliosis H3K27ac ChIP-seq (red) and mesenchymal stem cell–derived chondrocyte H3K4me1 (green) enrichment at the rs9496392 enhancer (highlighted in yellow). (*E*) Predicted disruption of transcription factor binding motifs. Error bars indicate mean ± SD.

The rs9496392 variant is located ∼32 kb downstream from the *ADGRG6* gene and coincides with H3K27ac enrichment in AIS patient cartilage (Makki et al. 2021) and with H3K4me1 in mesenchymal stem cell–derived chondrocytes (Roadmap Epigenomics Consortium ID E049) (Fig. 4D; Kundaje et al. 2015). GTEx data identify this SNP as an expression quantitative trait locus (eQTL) targeting the *ADGRG6* gene in lower leg skin, suprapubic skin, esophagus muscularis mucosa, gastrocnemius medialis, and subcutaneous adipose tissue (Boyle et al. 2012; Dong et al. 2023). As enhancers are tissue dependent and their target gene may vary between tissues, we wanted to confirm that rs9496392 interacts with *ADGRG6* in chondrocytes. Using published Hi-C data from the C28/I2 human chondrocyte cell line (Thulson et al. 2022), which has a similar gene expression profile to the TC28a2 cell line (Finger et al. 2003), we confirmed that the region flanking rs9496392 has some degree of interaction with the *ADGRG6* promoter (Supplemental Fig. S10). We next wanted to determine potential upstream regulators of this enhancer. The genomic area encompassing rs9496392 displayed enrichment of several TFs by ChIP across several biosamples (Boyle et al. 2012), indicating that the enhancer may be bound by these TFs. ChIP-seq data showing peaks at rs9496392 were available for CTCF in 15 biosamples, MAFK in five biosamples, NFE2L2 in three biosamples, RAD21 in two biosamples, and several other TFs across various other biosamples (Supplemental Table S9). Interestingly, motifbreakR analysis predicts that the AIS risk allele of rs9496392 (T) strongly disrupts TFBSs for SP1, PATZ1, and PRDM4 (Fig. 4E). These findings suggest that rs9496392 may affect expression of *ADGRG6* and other cartilage-related genes by altering TF binding affinity at this enhancer.

## Discussion

AIS is a common childhood disease that has significant impacts on quality of life; however, little is known about the underlying etiology. GWASs have provided a glimpse into potential underlying mechanisms, although a drawback of these studies is their reliance on association, which leaves the contribution and function of associated variants as an open question. Few AIS regulatory variants have been identified thus far, as traditional individual reporter assays are low throughput and time consuming and are biased owing to their reliance on correlative data to identify putative enhancers. Here we performed the first massively parallel assessment of AIS-associated variants to gain a better understanding of the genetic basis of AIS and the gene regulatory networks that may be perturbed in patients. To that end, we identified 1664 variants in high LD with 26 lead SNPs that were previously identified by GWASs and tested all possible alternate alleles to identify variants that differentially affect gene regulatory sequences in a high-throughput, unbiased manner.

In this work, we focused on identifying functional AIS variants that disrupt gene regulatory activity in chondrocytes, the primary cell type of cartilage, as cartilage has been widely implicated in AIS pathogenesis through studies in patients and model organisms (Karner et al. 2015; Ogura et al. 2015; Wise et al. 2020; Liu et al. 2021; Makki et al. 2021; Ushiki et al. 2024; Yu et al. 2024). We selected two chondrocyte cell lines, TC28a2 and SW1353, that are commonly used in the field of cartilage biology. We identified 659 unique genomic positions active in TC28a2 and 499 unique genomic positions active in SW1353; 886 of which were shared between both cell lines. This is the first functional identification of active enhancer elements in chondrocytes to be performed en masse, providing a new catalog of active enhancers for TC28a2 and SW1353.

Next, we identified CRSs by reference–alternate variant pairs to identify those with significant differences in transcription between the two alleles and with at least one allele being significantly active in the cell line. Our analysis identified a list of 234 highly confident variant pairs with allele-dependent regulatory activity, of which 92 are present in our selected HapMap populations. The remaining significant variants are not present in the selected populations but have similar numbers to the observed alternate alleles at each stage of the analysis. The 92 identified regulatory variants are spread across 18 of the 26 AIS GWAS loci. Some of these loci have a known role in AIS pathogenesis, such as *ADGRG6* and *PAX1*, and identification of novel functional AIS-associated variants at these loci will open many avenues for future investigations into the role these enhancer variants play in gene regulation. Other functional variants are at loci with unknown roles in AIS pathogenesis, such as *CDH13* and *MIR4300HG*. Identification of functional variants at these loci will prompt new studies to investigate the mechanisms by which these loci affect AIS pathogenesis. Of the 92 regulatory variants, 79 are predicted to disrupt TFBS, which is critical for normal enhancer activity, and thus increase our understanding of potential mechanisms of effect for these variants. We attempted investigating the overlap of the 92 significant variants with markers for active enhancers, including ATAC-seq and H3K27ac ChIP-seq peaks, but as these data are not available for the two cell lines used in this study, we were unable to properly ascertain variant pairs overlapping ATAC or H3K27ac regions. Given the cell-specific context of epigenetic modifications, and the fact that epigenetic data from chondrocytes is largely lacking, generation of these data sets will be an important step toward understanding the regulatory mechanisms governing disorders that affect cartilage. Future studies will be aimed at directly linking functional variants in enhancer elements to effects on TF binding and to changes in target gene expression, thus elucidating gene regulatory networks underlying AIS pathogenesis.

Interestingly, one of the variants with differential regulatory activity in both cell lines (rs9496392) is located at the *ADGRG6* locus, which has a high relevance to cartilage and chondrocyte biology. In addition, the region flanking this SNP has chromatin modifications that are characteristic of enhancers (H3K27ac and H3K4me1) in cartilage and chondrocytes, is bound by several TFs in different tissues, and is predicted to have some interaction with the *ADGRG6* promoter. *ADGRG6* encodes an adhesion G protein–coupled receptor, previously called *GPR126*, that has established roles in chondrogenesis and chondrocyte homeostasis. Cartilage-specific knockout of *ADGRG6* in the osteochondral progenitor lineage and committed chondrocytes resulted in a postnatal scoliosis-like phenotype in mice that develops around the onset of puberty (Karner et al. 2015; Liu et al. 2021), whereas conditional deletion in osteoblasts resulted in reduced bone mineralization and body length (Sun et al. 2020). The novel regulatory variant (rs9496392) identified in our study is located downstream from the *ADGRG6* locus and is predicted to interact with the *ADGRG6* promoter. The risk allele of the variant is predicted to reduce affinity for several TFs including SP1, which has been shown to transactivate *COL2A1* expression (Ghayor et al. 2001; Chadjichristos et al. 2003). Recent studies have shown that ADGRG6 acts as an upstream regulator of COL2A1, among other ECM genes, in the postnatal mouse intervertebral disc. It is interesting to speculate that SP1 may regulate *ADGRG6* and that its dysregulation in AIS may contribute to compromised ECM integrity (Aceves et al. 2026).

Although this study identified a high-confidence set of AIS-associated regulatory variants with allele-dependent activity in chondrocyte-derived cells, a number of limitations warrant further investigation in future follow-up studies. First, because of the complexity and size of the variant library, at about 7 million associated barcodes with an average number of 735 barcodes per CRS, we observed lower integration rates than anticipated. This lower representation may reduce sensitivity to detect regulatory effects, particularly for variants with modest allelic effects or lower barcode support, and may therefore increase the false-negative rate. However, the significant findings reported here are supported by multiple empirical reproducibility metrics, including strong CRS-level replicate concordance for aggregated DNA counts, RNA counts, and RNA/DNA ratios, as well as high agreement between full-barcode and barcode-subsampled MPRAnalyze results. Thus, although improved integration efficiency or expanded coverage may identify additional disruptive variants in future studies, these quality-control analyses support the robustness of the significant CRS-level findings reported here. Second, although we have used genomic proximity to putatively annotate significantly disruptive variants with nearby genes, we recognize that proximity alone does not establish the affected gene or regulatory mechanism. Additional functional and mechanistic studies will be necessary to define the relevant downstream regulatory pathways.

To our knowledge, this is the first MPRA of AIS variants and the first MPRA to be performed in chondrocytes. This provides a ranked list of high-confidence functional AIS-associated variants with allele-specific regulatory ability, as well as a catalog of active gene regulatory elements in chondrocytes. In this study, we focused on chondrocytes owing to their known role in AIS pathogenesis through studies in patients and model organisms. Future studies will expand this analysis to other tissues relevant to AIS pathogenesis including bone, muscle, and sensory neurons. These findings will pave the way for the development of diagnostic and prognostic tests, as well as targeted therapies.

## Methods

### MPRA library design

The MPRA design included 26 lead SNP positions identified in previous GWASs (Wise et al. 2020). We then expanded the library to include 1638 additional variants that are in a LD greater than or equal to 0.7 with these lead SNPs in the CEU, CHB, and JPT HapMap3 populations (The 1000 Genomes Project Consortium et al. 2015). We included all four alleles for each of the SNPs, as well as only the reference and single alternate allele for indels. In total, the library includes 4708 variant pairs (1522 reference SNPs × 3 alternate alleles + 142 reference/alternate indels) creating a total of 6372 tested CRSs (4708 alternate alleles + 1644 reference alleles). For positive controls, we selected 1000 sequences centered on the 1000 most significant *q*-valued H3K27ac ChIP-seq peaks identified in spinal cartilage (NCBI BioProject [https://www.ncbi.nlm.nih.gov/bioproject/] accession number PRJNA625649) (Makki et al. 2021) as identified by MACS2 (Zhang et al. 2008). Peaks were then filtered down to 601 that are within 1 Mb of a transcription start site for a gene with a RNA-seq value of at least one TPM in either the TC28a2 or SW1353 cell line. Negative controls were generated by randomly selecting and scrambling 200 of the reference allele CRSs. Each CRS was generated as a 200 bp sequence with the allele of interest at base pair 101. For indels, base pair 101 is the first base of the added sequence.

### lentiMPRA library cloning and preparation of association sequencing library

The CRS library was cloned according to the protocol described by Gordon et al. (2020). In brief, the CRS library was synthesized by Agilent. Vector homology overhangs, a minimal promoter, sequencing adaptors, and 15 bp random barcodes were added to the CRSs by sequential rounds of PCR. CRS inserts were then cloned into linearized pLS-SceI (Addgene 137725) using the NEBuilder HiFi DNA assembly master mix (NEB E2621S), electroporated into 10-beta electrocompetent cells (NEB C3020K), and plated on carbenicillin selection plates overnight at 37°C. Colonies were pooled, and plasmids were isolated by midiprep (Qiagen 12945). To associate barcodes with CRSs, the CRS-barcode region was amplified by PCR using primers appended with Illumina flowcell adapters. Association sequencing was carried out on an Illumina NovaSeq X (PE150) with custom primers (Supplemental Table S10).

### Barcode–CRS association

DNA reads from the association library were processed using the MPRAflow pipeline (Gordon et al. 2020) and aligned to the CRSs using Bowtie 2 (Langmead and Salzberg 2012) with the “very sensitive” present parameters. Sequences were retained only if they contained an exact match to the CRS (‐‐cigar 270 M) and that barcode uniquely mapped to that CRS at least 70% of the time (‐‐min-frac 0.70). Additionally, barcodes were associated with a CRS only if there were at least three instances of that barcode associating to that CRS (min-cov 3). We observed 30,673,678 barcodes across all association sequences and confidently associated 7,361,694 barcodes (24%) to CRSs at an average of 735 barcodes per CRS.

### Cell lines

The TC28a2 cell line was acquired from Dr. Miguel Otero, courtesy of Dr. Mary Goldring. SW1353 (HTB-94) and 293T (CRL-3216) cells were purchased from ATCC. All cells were cultured in DMEM (Gibco 11995-065) supplemented with 10% fetal bovine serum (Corning MT35010CV) and 1% penicillin–streptomycin (Gibco 15140122) and were maintained in a 5% CO_2_ humidified atmosphere.

### Lentivirus packaging

293T cells were cotransfected with the CRS library, pMD2.G (Addgene 12259), and psPAX2 (Addgene 12260) plasmids using the EndoFectin Lenti transfection reagent (GeneCopoeia EF001) per the manufacturer's protocol. The cell culture media were replaced with fresh media supplemented with 5% FBS, 1% penicillin–streptomycin, and 1× ViralBoost reagent (Alstem VB100) after 8 h. After 48 h, media were collected and filtered through a 0.45 µm PES filter to remove cellular debris, and viral particles were concentrated using the Lenti-X concentrator reagent (Takara Bio 631232). To determine the virus titer, cells were seeded so as to yield 80% confluency the next day in a 24-well plate; then the media were replaced with DMEM supplemented with polybrene (Sigma-Aldrich TR-1003-G), and increasing amounts of virus was added to each well (0, 1, 2, 4, 8, 16, 32, 64 µL). Media were changed after 24 h, and genomic DNA was extracted after 48 h using the Wizard SV genomic DNA purification kit (Promega A2361). The titer and multiplicity of infection (MOI) were determined by qPCR using the ΔCt method with primers targeting viral WPRE, plasmid backbone, and human LP34 genomic DNA.

### Lentiviral infection and preparation of barcode sequencing library

Lentivirus infection of TC28a2 and SW1353 cell lines were carried out in triplicate. Cells were seeded in 15 cm dishes so as to yield 80% confluency the next day (about 3.9 million TC28a2 cells or 2.7 million SW1353), and then the following day media were replaced with media containing polybrene. To maintain library complexity, cells were transduced at a high MOI (∼100), as determined by prior titration for optimal viability (Gordon et al. 2020). With a total of ∼3.9 × 10^6^ TC28a2) and ∼2.7 × 10^6^ (SW1353) cells per replicate and a functional titer of 0.5–1 × 10^6^ TU/µL, we achieved about 270 million to 390 million integrations per sample. Based on the full barcode library complexity, this corresponds to an average representation of approximately nine to 13 integrations per barcode across the input library. The next day, fresh media were added, and 48 h after infection, DNA and RNA were simultaneously extracted using the AllPrep DNA/RNA mini kit (Qiagen 80204). Cells were lysed in RLT Plus lysis buffer supplemented with 2-mercaptoethanol, scraped off the dish, and homogenized using a 3 mL syringe and 20-gauge needle. RNA was treated with the RNase-free DNase set (Qiagen 79256) according to the manufacturer's protocol, followed by treatment with the TURBO DNase kit (Invitrogen AM1907) according to the manufacturer's protocol for rigorous DNase treatment. RNA was then reverse-transcribed using the SuperScript II reverse transcriptase (Invitrogen 18064-071) and a primer downstream from the barcode containing an UMI and an Illumina P7 flowcell sequence.

To generate the barcode sequencing library, a P5 flowcell sequence and unique sample indexes were added to each DNA/RNA sample from each replicate by three cycles of PCR. Then, a second round of PCR was carried out with P5 and P7 primers. The number of PCR cycles for the second-round PCR was determined by qPCR, that is, the cycle in which the amplification curve nearly plateaus. The second-round PCR product was gel-extracted and purified using the NucleoSpin gel & PCR clean-up mini kit (Macherey-Nagel 740609.50) and quantified using a Qubit 3.0 fluorometer with the high-sensitivity dsDNA kit (Invitrogen Q33230). Finally, DNA and cDNA libraries were pooled in a 1:3 ratio before sequencing. Sequencing was performed on an Illumina NextSeq (PE75) with custom primers corresponding to the barcode, sample index, and UMI (Supplemental Table S10).

### Barcode counting

DNA and RNA reads from the assay were processed using the MPRAflow pipeline (Gordon et al. 2020) to obtain the number of barcode counts to be attributed to associated CRSs. Reads were required to exactly match the barcode (bc-length 15). CRSs were retained for further analysis only if they contained at least 10 associated barcodes with observable DNA and RNA reads (‐‐thresh 10; ‐‐merge_intersect TRUE). The TC28a2 cell line contained counts for 15,386,987 total barcodes, including 4,878,603 (66.27%) confidently assigned barcodes. The SW1353 cell line contained counts for 17,264,142 total barcodes, including 4,563,152 (61.98%) confidently assigned barcodes.

### Quantitative analysis of transcriptional activity

Quantification of a CRS transcriptional activity was calculated utilizing MPRAnalyze (Ashuach et al. 2019). Briefly, MPRAnalyze first calculates library wide normalization factors and then fits generalized linear models (GLMs) to the barcode counts. The program fits a GLM to the latent construct estimates and observed DNA counts as well as fits a second nested GLM to the latent rate of transcription from the latent construct estimates and the observed RNA counts. MPRAnalyze then optimizes the GLM's via a gamma likelihood maximization of the DNA counts and a negative binomial likelihood maximization of the RNA counts. Transcriptional activity of the sequences is calculated utilizing the analyzeQuantification command to compare all sequences to the activity of the negative controls, while normalizing for library-wide and replicate variation. This outputs an estimated transcriptional “alpha” value and a median absolute deviation (MAD) *Z*-score-based *P*-value comparing the alpha value to the median alpha values of the negative control sequences. CRSs are reported as significantly active compared with negative controls with a *P*-value ≤ 0.05. Transcriptional activity variation between reference and alternate allele pairs are calculated utilizing the analyzeComparative command of MPRAnalyze. This command provides a more sensitive calculation between the reference and alternate sequences than only comparing the alpha values from the analyzeQuantification command. This command includes the same normalizations as in the analyzeQuantification command but also incorporates the normalization of the different barcodes associated with the reference or alternate allele in the sequence pair. Additionally, the command normalized the overall difference between the reference and alternate allele to the RNA GLM to act as the null hypothesis. This command outputs the same alpha values as before, but now, the *P*-value is the significant difference between the reference and alternate allele, while accounting for each allele's difference from the negative control. Alternate alleles have a significant difference compared with their reference allele sequence pair with a FDR ≤0.10. As the model fits the numerous barcode counts to the GLM models and reports the fitted alpha values, standard error metrics are not typically reported. Rather, they are incorporated into the *P*-values comparing the activity of the allele to either the negative controls or the other allele in the variant pair.

Because of the large complexity of the library, we were computationally unable to use all counted barcodes for the nested GLM calculation of differential allelic expression between variant pairs. To account for this, we selected one representative variant pair at each of the loci tested and generated the MPRAnalyze comparative results on those variant pairs. We then subsetted the full variant pair table to only analyze 500 barcodes per CRS, which we were able to analyze with our available computational hardware. We then compared the results between the two approaches for variant pairs analyzed in both. We identified a 99.5% and 99.8% correlation of log fold change between alleles for TC28a2 and SW1353, respectively, and a high correlation between the FDR values calculated, with only one out of a subset of variants pairs tested using all available barcodes, losing significance. With this high correlation of both log fold change and FDR, we felt confident to continue the analysis, calculating differential allelic expression using only 500 barcodes per CRS.

To generate a final set of variants that have a high confidence of transcriptional disruption, we overlap the results of the analyzeQuantification and the analyzeComparative. For a variant pair to be considered high confidence, the alleles must be significantly different from each other as reported by the analyzeComparative analysis. To add confidence that the disruption is having an effect in the cell line, we also require the variant pair to have at least one of the two alleles be significantly active compared with the negative controls in the analyzeQuantification analysis. This prevents any variant pairs that are significantly different from each other, but neither have an effect in the cell line as the transcription rates are at the same level as the negative controls.

### Luciferase validation assays

CRSs (Supplemental Table S11) were amplified from human genomic DNA (Promega G304A) by PCR, cloned into pGL4.23[luc2/mP] (Promega E8411) using the NEBuilder HiFi DNA assembly kit (NEB E2621S), and then transformed into DH5-alpha competent cells (NEB C2987H) and plated on carbenicillin selection plates overnight at 37°C. Successful insertion of the CRS was verified by colony PCR; plasmids were isolated by miniprep (Macherey-Nagel 740490.50) and sequenced. The alternate SNP alleles were introduced by site-directed mutagenesis using a phusion site-directed mutagenesis kit (Thermo Fisher Scientific F541). Cell lines were plated in 24-well plates to yield 80% confluency the next day and then transfected with 900 ng of the CRS plasmid and 100 ng of pGL4.74[hR/luc] (Promega E6921) using X-tremeGENE HP DNA transfection reagent (Roche 6366244001) according to the manufacturer's protocol. After 48 hours post-transfection, luciferase activity was assayed using the dual luciferase reporter assay system (Promega E1980) according to the manufacturer's protocol on a Glomax multidetection system plate reader (Promega 9301-010).

CRS activity was measured in comparison to cells transfected with empty vector negative control plasmids (pGL4.23) with at least six biological replicates for each construct. The statistical significance between CRSs and negative controls was calculated by one-way ANOVA with the Dunnett's test to account for multiple comparisons, whereas statistical significance between SNP alleles of the same CRS was calculated by an unpaired *t*-test with Benjamini–Krieger–Yekutieli correction for multiple comparisons.

### TF binding disruption predictions

TFBS disruptions were predicted using the program MotifBreakR (Coetzee et al. 2015). Briefly, MotifBreakR utilizes a position weight matrix from the ENCODE database of TFBSs to predict if a variant causes a neutral, weak, or strong disruption to a predicted binding site. In our study, we only included disruptions to binding sites if the significance *P*-value of disruption was ≤1 × 10^−4^, and the disruption to the binding site was listed as weak or strong. For our analysis, we further filtered any binding site that was for a TF not expressed (<1 TPM) in the cell line that the variant pair was found significant in.

### Generation of TC28a2 RNA-seq data and processing of TC28a2 and SW1353 RNA-seq

Total RNA from the TC28a2 cell line was isolated using the Qiagen RNeasy mini kit, and mRNA was selected for using poly(T) oligo-attached magnetic beads. First-strand cDNA was synthesized using random hexamers, followed by second-strand cDNA synthesis. Paired-end sequencing was carried out on an Illumina NovaSeq6000. Raw RNA-seq FASTQ files for the SW1353 cell line were downloaded from the NCBI Gene Expression Omnibus (GEO; https://www.ncbi.nlm.nih.gov/geo/) database under accession number GSE176234.

For processing the sequenced FASTQ files, we utilized nf-core's standardized RNA-seq pipeline (version 3.12.0) (Ewels et al. 2020) with the following additional parameters. For the Trim Galore! Step, we include the following on top of the default parameters: –trim-n to remove trailing N's on either side of the read, –length 40 to remove reads with a trimmed size less than 40 bases, –quality 20 to remove ends of reads with below 20 Phred scores, and –clip_R1 5 and clip_R2 5 to trim the first five bases off of the 5′ and 3′ end of the reads. Additionally, we include –min_trimmed_reads 5000 to remove any samples with fewer than 5000 reads after trimming. We then include –skip_alignment, –skip_deseq2_qc, and –psuedo_aligner salmon with an igenomes reference file set to call TPM counts for genes in the cell lines. The generated file is a two-column table with HGNC gene symbols and the TPM value. We then remove any gene symbols with a TPM value less than one to get a list of genes active in the SW1353 and TC28a2 cell lines, respectively.

## Data access

All raw and processed sequencing data generated in this study have been submitted to the NCBI Gene Expression Omnibus (GEO; https://www.ncbi.nlm.nih.gov/geo/) under accession numbers GSE316848 (MPRA) and GSE316849 (TC28a2 RNA-seq).

## Supplemental Material

Supplement 1

Supplement 2

Supplement 3

Supplement 4

Supplement 5

Supplement 6

## References

[GR281888RamC1] The 1000 Genomes Project Consortium, Abecasis GR, Altshuler DM, Durbin RM, Bentley DR, Chakravarti A, Clark AG, Donnelly P, Eichler EE, Flicek P, 2015. A global reference for human genetic variation. Nature 526: 68–74. 10.1038/nature15393 PMC4750478 26432245

[GR281888RamC2] Aceves V, Xu Z, Zhang C-H, Kim J, Ito J, Mathers K, Lassar A, Liu Z, Gray R. 2026. Genetic interaction between Adgrg6 and Sox9 reveals a feedforward mechanism for postnatal spinal stability. bioRxiv 10.64898/2026.04.06.716681

[GR281888RamC3] Andersen MO, Thomsen K, Kyvik KO. 2007. Adolescent idiopathic scoliosis in twins: a population-based survey. Spine 32: 927–930. 10.1097/01.brs.0000259865.08984.00 17426641

[GR281888RamC4] Ashuach T, Fischer DS, Kreimer A, Ahituv N, Theis FJ, Yosef N. 2019. MPRAnalyze: statistical framework for massively parallel reporter assays. Genome Biol 20: 183. 10.1186/s13059-019-1787-z PMC6717970 31477158

[GR281888RamC5] Bachmann-Gagescu R, Phelps IG, Stearns G, Link BA, Brockerhoff SE, Moens CB, Doherty D. 2011. The ciliopathy gene *cc2d2a* controls zebrafish photoreceptor outer segment development through a role in Rab8-dependent vesicle trafficking. Hum Mol Genet 20: 4041–4055. 10.1093/hmg/ddr332 PMC3177654 21816947

[GR281888RamC6] Barat-Houari M, Dumont B, Fabre A, Them FTM, Alembik Y, Alessandri J-L, Amiel J, Audebert S, Baumann-Morel C, Blanchet P, 2016. The expanding spectrum of *COL2A1* gene variants IN 136 patients with a skeletal dysplasia phenotype. Eur J Hum Genet 24: 992–1000. 10.1038/ejhg.2015.250 PMC5070901 26626311

[GR281888RamC7] Becker-Heck A, Zohn IE, Okabe N, Pollock A, Lenhart KB, Sullivan-Brown J, McSheene J, Loges NT, Olbrich H, Haeffner K, 2011. The coiled-coil domain containing protein CCDC40 is essential for motile cilia function and left-right axis formation. Nat Genet 43: 79–84. 10.1038/ng.727 PMC3132183 21131974

[GR281888RamC8] Bieder A, Chandrasekar G, Wason A, Erkelenz S, Gopalakrishnan J, Kere J, Tapia-Páez I. 2023. Genetic and protein interaction studies between the ciliary dyslexia candidate genes *DYX1C1* and *DCDC2*. BMC MolCell Biol 24: 20. 10.1186/s12860-023-00483-4 PMC10224228 37237337

[GR281888RamC9] Blecher R, Krief S, Galili T, Biton IE, Stern T, Assaraf E, Levanon D, Appel E, Anekstein Y, Agar G, 2017. The proprioceptive system masterminds spinal alignment: insight into the mechanism of scoliosis. Dev Cell 42: 388–399.e3. 10.1016/j.devcel.2017.07.022 28829946

[GR281888RamC10] Boyle AP, Hong EL, Hariharan M, Cheng Y, Schaub MA, Kasowski M, Karczewski KJ, Park J, Hitz BC, Weng S, 2012. Annotation of functional variation in personal genomes using RegulomeDB. Genome Res 22: 1790–1797. 10.1101/gr.137323.112 PMC3431494 22955989

[GR281888RamC11] Buchan JG, Alvarado DM, Haller GE, Cruchaga C, Harms MB, Zhang T, Willing MC, Grange DK, Braverman AC, Miller NH, 2014a. Rare variants in FBN1 and FBN2 are associated with severe adolescent idiopathic scoliosis. Hum Mol Genet 23: 5271–5282. 10.1093/hmg/ddu224 PMC4159151 24833718

[GR281888RamC12] Buchan JG, Gray RS, Gansner JM, Alvarado DM, Burgert L, Gitlin JD, Gurnett CA, Goldsmith MI. 2014b. Kinesin family member 6 (Kif6) is necessary for spine development in zebrafish. Dev Dyn 243: 1646–1657. 10.1002/dvdy.24208 PMC6207368 25283277

[GR281888RamC13] Chadjichristos C, Ghayor C, Kypriotou M, Martin G, Renard E, Ala-Kokko L, Suske G, de Crombrugghe B, Pujol J-P, Galéra P. 2003. Sp1 and Sp3 transcription factors mediate interleukin-1 beta down-regulation of human type II collagen gene expression in articular chondrocytes. J Biol Chem 278: 39762–39772. 10.1074/jbc.M303541200 12888570

[GR281888RamC14] Coetzee SG, Coetzee GA, Hazelett DJ. 2015. *motifbreakR*: an R/Bioconductor package for predicting variant effects at transcription factor binding sites. Bioinformatics 31: 3847–3849. 10.1093/bioinformatics/btv470 PMC4653394 26272984

[GR281888RamC15] de Azevedo GBL, Perini JA, Araújo Junior AEP, Moliterno LAM, Andrande RM, Guimarães JAM, Defino HLA. 2022. Association of FBN1 polymorphism with susceptibility of adolescent idiopathic scoliosis: a case-control study. BMC Musculoskel Disord 23: 430. 10.1186/s12891-022-05370-1 PMC9077855 35526034

[GR281888RamC16] Decourtye L, McCallum-Loudeac JA, Zellhuber-McMillan S, Young E, Sircombe KJ, Wilson MJ. 2022. Characterization of a novel Lbx1 mouse loss of function strain. Differentiation 123: 30–41. 10.1016/j.diff.2021.12.001 34906895

[GR281888RamC17] Dong S, Zhao N, Spragins E, Kagda MS, Li M, Assis P, Jolanki O, Luo Y, Cherry JM, Boyle AP, 2023. Annotating and prioritizing human non-coding variants with RegulomeDB v.2. Nat Genet 55: 724–726. 10.1038/s41588-023-01365-3 PMC10989417 37173523

[GR281888RamC18] Ewels PA, Peltzer A, Fillinger S, Patel H, Alneberg J, Wilm A, Garcia MU, Di Tommaso P, Nahnsen S. 2020. The Nf-core framework for community-curated bioinformatics pipelines. Nat Biotechnol 38: 276–278. 10.1038/s41587-020-0439-x 32055031

[GR281888RamC19] Fan Y-H, Song Y-Q, Chan D, Takahashi Y, Ikegawa S, Matsumoto M, Kou I, Cheah KSE, Sham P, Cheung KMC, 2012. SNP rs11190870 near LBX1 is associated with adolescent idiopathic scoliosis in southern Chinese. J Hum Genet 57: 244–246. 10.1038/jhg.2012.11 22301463

[GR281888RamC20] Finger F, Schörle C, Zien A, Gebhard P, Goldring MB, Aigner T. 2003. Molecular phenotyping of human chondrocyte cell lines T/C-28a2, T/C-28a4, and C-28/I2. Arthritis Rheum 48: 3395–3403. 10.1002/art.11341 14673991

[GR281888RamC21] Ghayor C, Chadjichristos C, Herrouin J-F, Ala-Kokko L, Suske G, Pujol J-P, Galéra P. 2001. SP3 represses the SP1-mediated transactivation of the human *COL2A1* gene in primary and de-differentiated chondrocytes. J Biol Chem 276: 36881–36895. 10.1074/jbc.M105083200 11447232

[GR281888RamC22] Gordon MG, Inoue F, Martin B, Schubach M, Agarwal V, Whalen S, Feng S, Zhao J, Ashuach T, Ziffra R, 2020. lentiMPRA and MPRAflow for high-throughput functional characterization of gene regulatory elements. Nat Protoc 15: 2387–2412. 10.1038/s41596-020-0333-5 PMC7550205 32641802

[GR281888RamC23] Grauers A, Danielsson A, Karlsson M, Ohlin A, Gerdhem P. 2013. Family history and its association to curve size and treatment in 1,463 patients with idiopathic scoliosis. Eur Spine J 22: 2421–2426. 10.1007/s00586-013-2860-z PMC3886503 23801015

[GR281888RamC24] Gray RS, Wilm TP, Smith J, Bagnat M, Dale RM, Topczewski J, Johnson SL, Solnica-Krezel L. 2014. Loss of Col8a1a function during zebrafish embryogenesis results in congenital vertebral malformations. Dev Biol 386: 72–85. 10.1016/j.ydbio.2013.11.028 PMC3938106 24333517

[GR281888RamC25] Gray RS, Gonzalez R, Ackerman SD, Minowa R, Griest JF, Bayrak MN, Troutwine B, Canter S, Monk KR, Sepich DS, 2021. Postembryonic screen for mutations affecting spine development in zebrafish. Dev Biol 471: 18–33. 10.1016/j.ydbio.2020.11.009 PMC10785604 33290818

[GR281888RamC26] Grimes DT, Boswell CW, Morante NFC, Henkelman RM, Burdine RD, Ciruna B. 2016. Zebrafish models of idiopathic scoliosis link cerebrospinal fluid flow defects to spine curvature. Science 352: 1341–1344. 10.1126/science.aaf6419 PMC5574193 27284198

[GR281888RamC27] Haller G, Alvarado D, Mccall K, Yang P, Cruchaga C, Harms M, Goate A, Willing M, Morcuende JA, Baschal E, 2016. A polygenic burden of rare variants across extracellular matrix genes among individuals with adolescent idiopathic scoliosis. Hum Mol Genet 25: 202–209. 10.1093/hmg/ddv463 PMC4690498 26566670

[GR281888RamC28] Haller G, McCall K, Jenkitkasemwong S, Sadler B, Antunes L, Nikolov M, Whittle J, Upshaw Z, Shin J, Baschal E, 2018. A missense variant in SLC39A8 is associated with severe idiopathic scoliosis. Nat Commun 9: 4171. 10.1038/s41467-018-06705-0 PMC6177404 30301978

[GR281888RamC29] Hayes M, Gao X, Yu LX, Paria N, Henkelman RM, Wise CA, Ciruna B. 2014. Ptk7 mutant zebrafish models of congenital and idiopathic scoliosis implicate dysregulated Wnt signalling in disease. Nat Commun 5: 4777. 10.1038/ncomms5777 PMC4155517 25182715

[GR281888RamC30] Henry SP, Liang S, Akdemir KC, de Crombrugghe B. 2012. The postnatal role of Sox9 in cartilage. J Bone Miner Res 27: 2511–2525. 10.1002/jbmr.1696 PMC3502666 22777888

[GR281888RamC31] Hoelen T-CA, Willems PC, Arts JJ, van Mastrigt G, Evers S. 2023. The economic and societal burden associated with adolescent idiopathic scoliosis: a burden-of-disease study protocol. N Am Spine Soc J 14: 100231. 10.1016/j.xnsj.2023.100231 PMC10333714 37440982

[GR281888RamC32] Hoornaert KP, Vereecke I, Dewinter C, Rosenberg T, Beemer FA, Leroy JG, Bendix L, Björck E, Bonduelle M, Boute O, 2010. Stickler syndrome caused by *COL2A1* mutations: genotype–phenotype correlation in a series of 100 patients. Eur J Hum Genet 18: 872–880. 10.1038/ejhg.2010.23 PMC2987380 20179744

[GR281888RamC33] Inoue F, Kircher M, Martin B, Cooper GM, Witten DM, McManus MT, Ahituv N, Shendure J. 2017. A systematic comparison reveals substantial differences in chromosomal versus episomal encoding of enhancer activity. Genome Res 27: 38–52. 10.1101/gr.212092.116 PMC5204343 27831498

[GR281888RamC34] Jaffe KM, Grimes DT, Schottenfeld-Roames J, Werner ME, Ku T-SJ, Kim SK, Pelliccia JL, Morante NFC, Mitchell BJ, Burdine RD. 2016. *c21orf59/kurly* controls both cilia motility and polarization. Cell Rep 14: 1841–1849. 10.1016/j.celrep.2016.01.069 PMC4775428 26904945

[GR281888RamC35] Karner CM, Long F, Solnica-Krezel L, Monk KR, Gray RS. 2015. *Gpr126/Adgrg6* deletion in cartilage models idiopathic scoliosis and pectus excavatum in mice. Hum Mol Genet 24: 4365–4373. 10.1093/hmg/ddv170 PMC4492399 25954032

[GR281888RamC36] Khanshour AM, Kou I, Fan Y, Einarsdottir E, Makki N, Kidane YH, Kere J, Grauers A, Johnson TA, Paria N, 2018. Genome-wide meta-analysis and replication studies in multiple ethnicities identify novel adolescent idiopathic scoliosis susceptibility loci. Hum Mol Genet 27: 3986–3998. 10.1093/hmg/ddy306 PMC6488972 30395268

[GR281888RamC37] Klein JC, Keith A, Rice SJ, Shepherd C, Agarwal V, Loughlin J, Shendure J. 2019. Functional testing of thousands of osteoarthritis-associated variants for regulatory activity. Nat Commun 10: 2434. 10.1038/s41467-019-10439-y PMC6547687 31164647

[GR281888RamC38] Klein JC, Agarwal V, Inoue F, Keith A, Martin B, Kircher M, Ahituv N, Shendure J. 2020. A systematic evaluation of the design and context dependencies of massively parallel reporter assays. Nat Methods 17: 1083–1091. 10.1038/s41592-020-0965-y PMC7727316 33046894

[GR281888RamC39] Kou I, Takahashi Y, Johnson TA, Takahashi A, Guo L, Dai J, Qiu X, Sharma S, Takimoto A, Ogura Y, 2013. Genetic variants in GPR126 are associated with adolescent idiopathic scoliosis. Nat Genet 45: 676–679. 10.1038/ng.2639 23666238

[GR281888RamC40] Kou I, Watanabe K, Takahashi Y, Momozawa Y, Khanshour A, Grauers A, Zhou H, Liu G, Fan YH, Takeda K, 2018. A multi-ethnic meta-analysis confirms the association of rs6570507 with adolescent idiopathic scoliosis. Sci Rep 8: 11575. 10.1038/s41598-018-29011-7 PMC6070519 30069010

[GR281888RamC41] Kou I, Otomo N, Takeda K, Momozawa Y, Lu H-F, Kubo M, Kamatani Y, Ogura Y, Takahashi Y, Nakajima M, 2019. Genome-wide association study identifies 14 previously unreported susceptibility loci for adolescent idiopathic scoliosis in Japanese. Nat Commun 10: 3685. 10.1038/s41467-019-11596-w PMC6695451 31417091

[GR281888RamC42] Kundaje A, Meuleman W, Ernst J, Bilenky M, Yen A, Heravi-Moussavi A, Kheradpour P, Zhang Z, Wang J, Ziller MJ, 2015. Integrative analysis of 111 reference human epigenomes. Nature 518: 317–330. 10.1038/nature14248 PMC4530010 25693563

[GR281888RamC43] Langmead B, Salzberg SL. 2012. Fast gapped-read alignment with Bowtie 2. Nat Methods 9: 357–359. 10.1038/nmeth.1923 PMC3322381 22388286

[GR281888RamC44] Liu Z, Hussien AA, Wang Y, Heckmann T, Gonzalez R, Karner CM, Snedeker JG, Gray RS. 2021. An adhesion G protein-coupled receptor is required in cartilaginous and dense connective tissues to maintain spine alignment. eLife 10: e67781. 10.7554/eLife.67781 PMC8328515 34318745

[GR281888RamC45] Londono D, Kou I, Johnson TA, Sharma S, Ogura Y, Tsunoda T, Takahashi A, Matsumoto M, Herring JA, Lam T-P, 2014. A meta-analysis identifies adolescent idiopathic scoliosis association with *LBX1* locus in multiple ethnic groups. J Med Genet 51: 401–406. 10.1136/jmedgenet-2013-102067 24721834

[GR281888RamC46] Makki N, Zhao J, Liu Z, Eckalbar WL, Ushiki A, Khanshour AM, Wu J, Rios J, Gray RS, Wise CA, 2021. Genomic characterization of the adolescent idiopathic scoliosis-associated transcriptome and regulome. Hum Mol Genet 29: 3606–3615. 10.1093/hmg/ddaa242 PMC7823110 33179741

[GR281888RamC47] Miyake A, Kou I, Takahashi Y, Johnson TA, Ogura Y, Dai J, Qiu X, Takahashi A, Jiang H, Yan H, 2013. Identification of a susceptibility locus for severe adolescent idiopathic scoliosis on chromosome 17q24.3. PLoS One 8: e72802. 10.1371/journal.pone.0072802 PMC3762929 24023777

[GR281888RamC48] Ogura Y, Kou I, Miura S, Takahashi A, Xu L, Takeda K, Takahashi Y, Kono K, Kawakami N, Uno K, 2015. A functional SNP in BNC2 is associated with adolescent idiopathic scoliosis. Am J Hum Genet 97: 337–342. 10.1016/j.ajhg.2015.06.012 PMC4573260 26211971

[GR281888RamC49] Ogura Y, Kou I, Takahashi Y, Takeda K, Minami S, Kawakami N, Uno K, Ito M, Yonezawa I, Kaito T, 2017. A functional variant in *MIR4300HG*, the host gene of microRNA MIR4300 is associated with progression of adolescent idiopathic scoliosis. Hum Mol Genet 26: 4086–4092. 10.1093/hmg/ddx291 29016859

[GR281888RamC50] Patten SA, Margaritte-Jeannin P, Bernard J-C, Alix E, Labalme A, Besson A, Girard SL, Fendri K, Fraisse N, Biot B, 2015. Functional variants of POC5 identified in patients with idiopathic scoliosis. J Clin Invest 125: 1124–1128. 10.1172/JCI77262 PMC4362221 25642776

[GR281888RamC51] Ramkhalawan D, Parrales P, Koesterich J, Montoya-Vazquez G, Cuna C, Kreimer A, McQuerry J, Ihnow S, Makki N. 2026. Integrated transcriptomic and functional analysis reveals tissue-specific molecular pathology in adolescent idiopathic scoliosis. HGG Adv 7: 100631. 10.1016/j.xhgg.2026.100631 PMC13316586 42244182

[GR281888RamC52] Rebello D, Wohler E, Erfani V, Li G, Aguilera AN, Santiago-Cornier A, Zhao S, Hwang SW, Steiner RD, Zhang TJi, 2023. COL11A2 as a candidate gene for vertebral malformations and congenital scoliosis. Hum Mol Genet 32: 2913–2928. 10.1093/hmg/ddad117 PMC10508038 37462524

[GR281888RamC53] Rogala EJ, Drummond DS, Gurr J. 1978. Scoliosis: incidence and natural history. A prospective epidemiological study. J Bone Joint Surg Am 60: 173–176. 641080

[GR281888RamC54] Sharma S, Gao X, Londono D, Devroy SE, Mauldin KN, Frankel JT, Brandon JM, Zhang D, Li Q-Z, Dobbs MB, 2011. Genome-wide association studies of adolescent idiopathic scoliosis suggest candidate susceptibility genes. Hum Mol Genet 20: 1456–1466. 10.1093/hmg/ddq571 PMC3049353 21216876

[GR281888RamC55] Sharma S, Londono D, Eckalbar WL, Gao X, Zhang D, Mauldin K, Kou I, Takahashi A, Matsumoto M, Kamiya N, 2015. A *PAX1* enhancer locus is associated with susceptibility to idiopathic scoliosis in females. Nat Commun 6: 6452. 10.1038/ncomms7452 PMC4365504 25784220

[GR281888RamC300] Shu T, Zhang D, Li J, Liu H, Cui L, Gu J, Wu L, Liu W, Wan J, Zheng X. 2025. m6A mRNA demethylase FTO promotes chondrogenic differentiation of human bone marrow mesenchymal stem cells by targeting SMAD3. Stem Cells 43: sxaf035. 10.1093/stmcls/sxaf035 40570063

[GR281888RamC56] Smits P, Lefebvre V. 2003. *Sox5* and *Sox6* are required for notochord extracellular matrix sheath formation, notochord cell survival and development of the nucleus pulposus of intervertebral discs. Development 130: 1135–1148. 10.1242/dev.00331 12571105

[GR281888RamC301] Später D, Hill TP, O'Sullivan RJ, Gruber M, Conner DA, Hartmann C. 2006. Wnt9a signaling is required for joint integrity and regulation of *Ihh* during chondrogenesis. Development 133: 3039–3049. 10.1242/dev.02471 16818445

[GR281888RamC57] Su Z, Yang Y, Wang S, Zhao S, Zhao H, Li X, Niu Y, Qiu G, Wu Z, Wu N, 2021. The mutational landscape of PTK7 in congenital scoliosis and adolescent idiopathic scoliosis. Genes (Basel) 12: 1791. 10.3390/genes12111791 PMC8619039 34828397

[GR281888RamC58] Sudmant PH, Rausch T, Gardner EJ, Handsaker RE, Abyzov A, Huddleston J, Zhang Y, Ye K, Jun G, Hsi-Yang Fritz M, 2015. An integrated map of structural variation in 2,504 human genomes. Nature 526: 75–81. 10.1038/nature15394 PMC4617611 26432246

[GR281888RamC59] Sun P, He L, Jia K, Yue Z, Li S, Jin Y, Li Z, Siwko S, Xue F, Su J, 2020. Regulation of body length and bone mass by Gpr126/Adgrg6. Sci Adv 6: eaaz0368. 10.1126/sciadv.aaz0368 PMC7083604 32219165

[GR281888RamC60] Takahashi Y, Kou I, Takahashi A, Johnson TA, Kono K, Kawakami N, Uno K, Ito M, Minami S, Yanagida H, 2011. A genome-wide association study identifies common variants near LBX1 associated with adolescent idiopathic scoliosis. Nat Genet 43: 1237–1240. 10.1038/ng.974 22019779

[GR281888RamC61] Tang NLS, Yeung H-Y, Hung VWY, Di Liao C, Lam T-P, Yeung H-M, Lee K-M, Ng BK-W, Cheng JC-Y. 2012. Genetic epidemiology and heritability of AIS: a study of 415 Chinese female patients. J Orthop Res 30: 1464–1469. 10.1002/jor.22090 22362628

[GR281888RamC62] Thulson E, Davis ES, D'Costa S, Coryell PR, Kramer NE, Mohlke KL, Loeser RF, Diekman BO, Phanstiel DH. 2022. 3D chromatin structure in chondrocytes identifies putative osteoarthritis risk genes. Genetics 222: iyac141. 10.1093/genetics/iyac141 PMC9713432 36099032

[GR281888RamC63] Tuncay IO, Lee EK, Gustafson A, Lee Y, Jung D, Koh J-Y, Lee W, Lee S, Shazand K. 2025. Whole genome sequencing in adolescent idiopathic scoliosis cohort implicates multiple biological pathways. NPJ Genom Med 10: 67. 10.1038/s41525-025-00520-5 PMC12514243 41073408

[GR281888RamC64] Ushiki A, Sheng RR, Zhang Y, Zhao J, Nobuhara M, Murray E, Ruan X, Rios JJ, Wise CA, Ahituv N. 2024. Deletion of *Pax1* scoliosis-associated regulatory elements leads to a female-biased tail abnormality. Cell Rep 43: 113907. 10.1016/j.celrep.2024.113907 PMC11005513 38461417

[GR281888RamC65] Van Gennip JLM, Boswell CW, Ciruna B. 2018. Neuroinflammatory signals drive spinal curve formation in zebrafish models of idiopathic scoliosis. Sci Adv 4: eaav1781. 10.1126/sciadv.aav1781 PMC6291318 30547092

[GR281888RamC66] Wang Y, Li M, Chan C-O, Yang G, Lam JC-K, Law BC-S, Lam T-P, Hung AL-H, Cheng JC-Y, Mok DK-W, 2022. Biological effect of dysregulated *LBX1* on adolescent idiopathic scoliosis through modulating muscle carbohydrate metabolism. Spine J 22: 1551–1565. 10.1016/j.spinee.2022.04.005 35460899

[GR281888RamC67] Wang W, Du X, Luo M, Yang N. 2023. FTO-dependent m^6^A regulates muscle fiber remodeling in an NFATC1–YTHDF2 dependent manner. Clin Epigenetics 15: 109. 10.1186/s13148-023-01526-5 PMC10320966 37408034

[GR281888RamC68] Weinstein SL. 2019. The natural history of adolescent idiopathic scoliosis. J Pediatr Orthop 39: S44–S46. 10.1097/BPO.0000000000001350 31169647

[GR281888RamC69] Wise C, Gao X, Shoemaker S, Gordon D, Herring J. 2008. Understanding genetic factors in idiopathic scoliosis, a complex disease of childhood. Curr Genomics 9: 51–59. 10.2174/138920208783884874 PMC2674301 19424484

[GR281888RamC70] Wise CA, Sepich D, Ushiki A, Khanshour AM, Kidane YH, Makki N, Gurnett CA, Gray RS, Rios JJ, Ahituv N, 2020. The cartilage matrisome in adolescent idiopathic scoliosis. Bone Res 8: 13. 10.1038/s41413-020-0089-0 PMC7062733 32195011

[GR281888RamC71] Wu Z, Wang Y, Dai Z, Qiu Y, Xu L, Zhu Z. 2019. Genetic variants of ABO and SOX6 are associated with adolescent idiopathic scoliosis in Chinese Han population. Spine 44: E1063–E1067. 10.1097/BRS.0000000000003062 30994600

[GR281888RamC72] Xu L, Feng Z, Dai Z, Qiu Y, Wu Z, Zhu Z. 2025. Novel rare variation of CCDC40 plays a role in the development of idiopathic scoliosis possibly via dysfunction of cilia motility. Spine J 25: 797–804. 10.1016/j.spinee.2024.12.011 39662682

[GR281888RamC73] Yang T, Jia Q, Guo H, Xu J, Bai Y, Yang K, Luo F, Zhang Z, Hou T. 2012. Epidemiological survey of idiopathic scoliosis and sequence alignment analysis of multiple candidate genes. Int Orthop 36: 1307–1314. 10.1007/s00264-011-1419-z PMC3353077 22183150

[GR281888RamC74] Yonezawa Y, Guo L, Kakinuma H, Otomo N, Yoshino S, Takeda K, Nakajima M, Shiraki T, Ogura Y, Takahashi Y, 2020. Identification of a functional susceptibility variant for adolescent idiopathic scoliosis that upregulates early growth response 1 (EGR1)-mediated UNCX expression. J Bone Miner Res 38: 144–153. 10.1002/jbmr.4738 36342191

[GR281888RamC75] Yu G, Wang L-G, Han Y, He Q-Y. 2012. clusterProfiler: an R package for comparing biological themes among gene clusters. Omics 16: 284–287. 10.1089/omi.2011.0118 PMC3339379 22455463

[GR281888RamC76] Yu H, Khanshour AM, Ushiki A, Otomo N, Koike Y, Einarsdottir E, Fan Y, Antunes L, Kidane YH, Cornelia R, 2024. Association of genetic variation in COL11A1 with adolescent idiopathic scoliosis. eLife 12: RP89762. 10.7554/eLife.89762 PMC10945706 38277211

[GR281888RamC77] Zhang Y, Liu T, Meyer CA, Eeckhoute J, Johnson DS, Bernstein BE, Nusbaum C, Myers RM, Brown M, Li W, 2008. Model-based Analysis of ChIP-Seq (MACS). Genome Biol 9: R137. 10.1186/gb-2008-9-9-r137 PMC2592715 18798982

[GR281888RamC78] Zhu Z, Tang NL-S, Xu L, Qin X, Mao S, Song Y, Liu L, Li F, Liu P, Yi L, 2015. Genome-wide association study identifies new susceptibility loci for adolescent idiopathic scoliosis in Chinese girls. Nat Commun 6: 8355. 10.1038/ncomms9355 PMC4595747 26394188

